# Supplementary material for: Transient reactivation of small ensembles of adult-born neurons during REM sleep supports memory consolidation in mice
Source: Nat Commun. 2025 Aug 5;16:7210. doi: 10.1038/s41467-025-62554-8 (PMC12325634; doi:10.1038/s41467-025-62554-8)
Supplement: Supplementary file 1 — Supplementary Information [file 41467_2025_62554_MOESM1_ESM.pdf]

Transient reactivation of small ensembles of adult-born neurons during REM sleep supports memory consolidation in mice

Supplementary Information

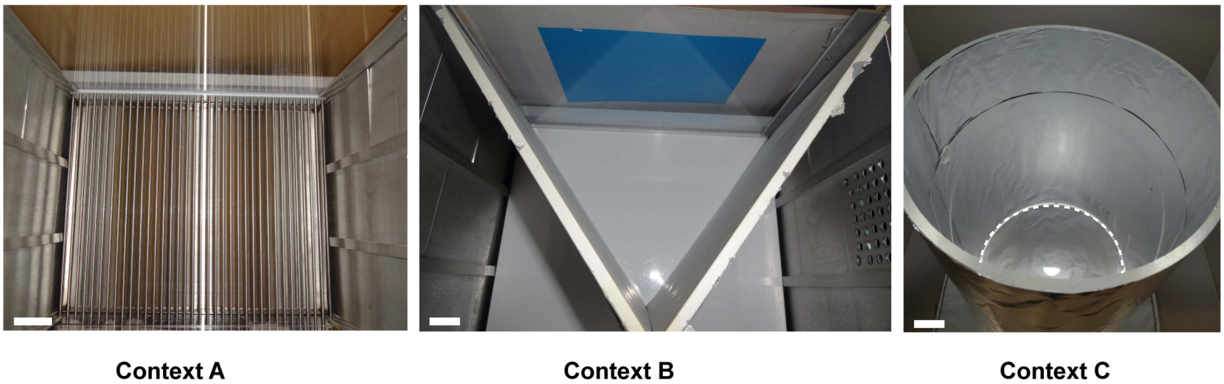

Supplementary Fig. 1. Images of the contexts used for behavioural testing. Broken line indicates the perimeter of the base of the chamber. Scale bars, 5 cm.

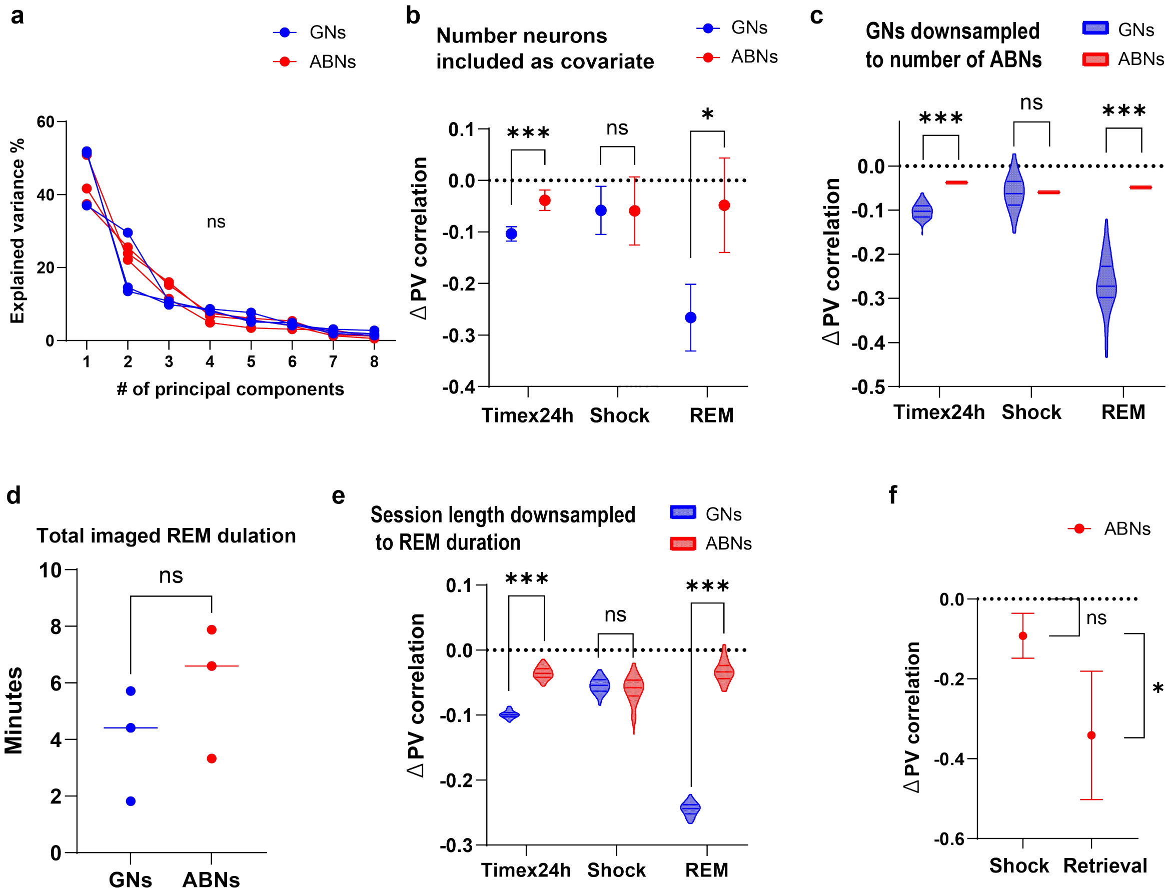

Supplementary Fig. 2, Ca^{2+} activity of active ABNs and GNs during conditioning and sleep, related to Fig. 1. (**a**) Data dimensionality between ABNs and GNs. Two-way repeated measures ANOVA with Sidak's multiple comparisons; p = 0.63 (**b, c,** and **e**) Effect of each variable on population vector (PV) correlations when (**b**) the number of neurons was included as a covariate, (**c**) the number of GNs was downsampled to match that of ABNs, and (**e**) each session was downsampled to the duration of REM sleep. (**d**) Amount of REM sleep in each group. (**f**) Change in PV correlation after shock and during memory retrieval. For all panels: GNs, n = 363 neurons in 3 mice; ABNs, n = 136 neurons in 3 mice. Data are presented as mean values and standard errors of the regression coefficients. ABNs adult-born neurons; GNs, granular neurons; PV, population vector; ns, not significant; *, $p < 0.05$, ***, $p < 0.001$.

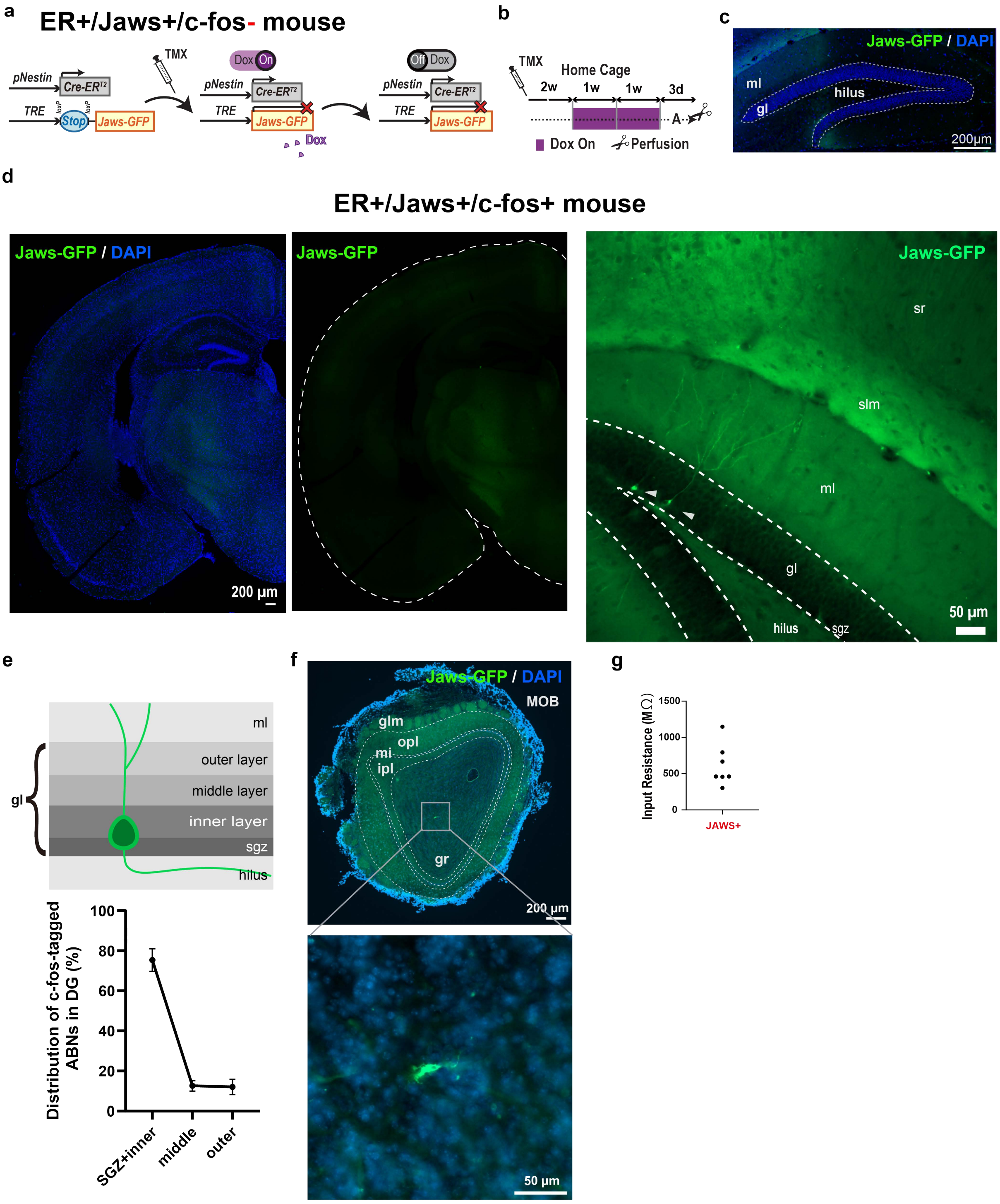

Supplementary Fig. 3. cfos-tagged ABNs. (**a**) Transgenic method for using nestin/jaws mice as a control for nestin/cfos/jaws mice. (**b**) Behavioural paradigm for quantifying Jaws-GFP expression. (**c**) Representative image of the DG in a nestin/jaws mouse. The experiment was repeated over 3 mice with similar results. (**d**) ER+/Jaws+/cfos+ mouse. Arrows, Jaws-GFP+ cells. The experiment was repeated over 3 mice with similar results. (**e**) Distribution of cfos-tagged ABNs in the DG (n = 207 cells from 8 mice; Error bars, SEM). (**f**) Jaws-GFP labeled ABNs were found in the olfactory bulb. Similar expressions were confirmed over 3 sections. (**g**) Input resistance of Jaws+ cells (n = 7 cells from 4 mice). TMX, tamoxifen; Dox, doxycycline; gl, granular cell layer; glm, glomerular layer; gr, granule layer; ipl, inner plexiform layer; mi, mitral layer; ml, molecular layer; MOB, main olfactory bulb; opl, outer

plexiform layer; sgz, subgranular zone; slm, stratum lacunosum-moleculare; sr, stratum radiatum; SEM, standard error of the mean.

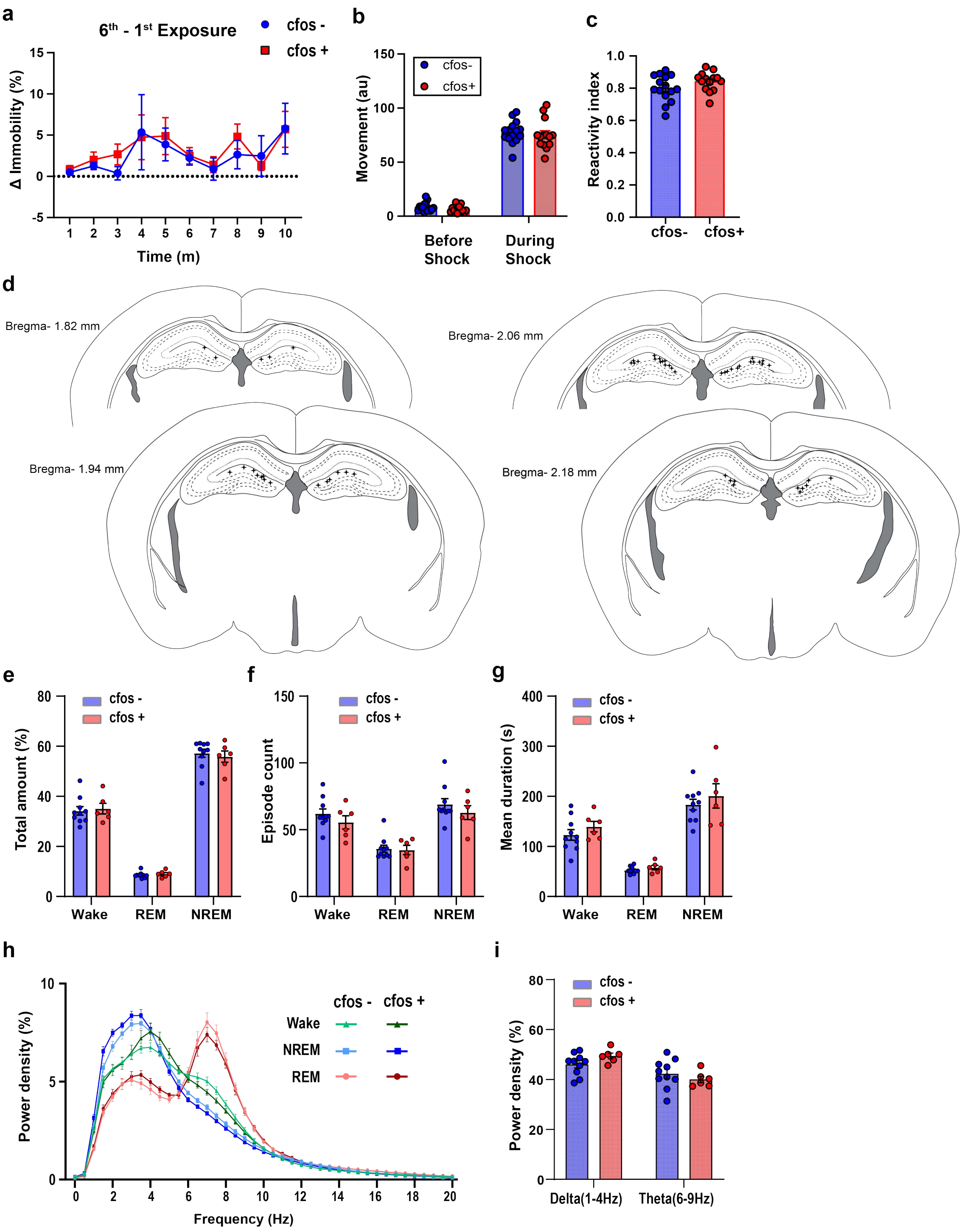

Supplementary Fig. 4. cfos- and cfos+ mice showed similar behaviour in basal activity, shock reactivity, and sleep architecture. (**a**) Change in immobility between first and last context A habituation sessions. Two-way repeated measures ANOVA, $p = 0.987$. (**b**) Shock reactivity. Two-way repeated measures ANOVA, $p = 0.940$. (**c**) Shock reactivity index. Two-tailed unpaired *t*-test, $p = 0.149$. cfos-, n = 15 mice; cfos+, n = 14 mice. au, arbitrary units. (**d**) Location of the optic fiber tip. Black cross marks indicate the tip of the optic fiber from >28 nestin/jaws and nestin/cfos/jaws mice in schematics of coronal sections of the mouse brain. (**e-i**) Sleep score under optogenetic silencing during REM sleep within 6 h after the conditioning

session. (**e**) Total amount of time, two-way repeated measures ANOVA, $p = 0.82$. (**f**) Episode counts, two-way repeated measures ANOVA, $p = 0.21$. (**g**) Mean episode duration, two-way repeated measures ANOVA, $p = 0.78$. (**h**) Fast Fourier transform analysis of EEG. (**i**) NREM sleep-delta (1-4 Hz) and REM sleep-theta (6-9 Hz) band, two-way repeated measures ANOVA, $p = 0.17$. cfos-, n = 10 mice; cfos+, n = 6 mice. Data are presented as mean values +/- SEM. Wake, wakefulness; NREM, Non-rapid eye movement sleep; REM, REM sleep; SEM, standard error of the mean.

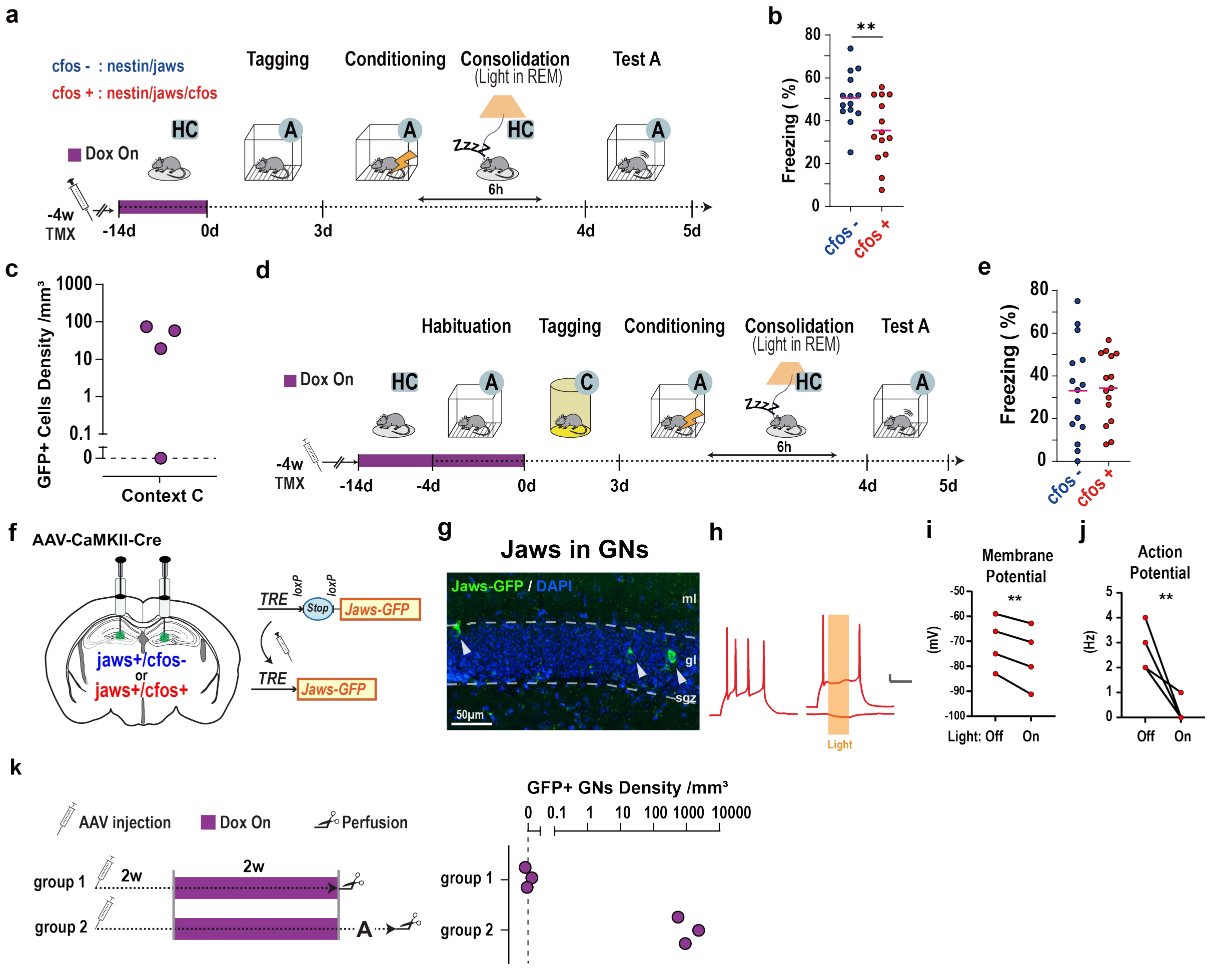

Supplementary Fig. 5. Silencing tagged ABNs during REM sleep impaired memory consolidation. (**a-b**) Data reproduced from a separate experiment using the same protocol as in Fig. 3B. (**a**) Protocol for silencing ABNs tagged in context A. (**b**) Freezing during the retrieval test. Two-tailed unpaired *t*-test, $p = 0.007$. cfos-, n = 14 mice; cfos+, n = 14 mice. Horizontal bars, mean.(**c-e**) Silencing context C-tagged neurons in REM sleep**. c.** Jaws-GFP cell density tagging in context C (n = 4 mice). (**d**) Protocol for silencing ABNs tagged in context A/C with context habituation. (**e**) Freezing in the retrieval test, two-tailed unpaired *t*-test, $p =$ 0.87. cfos-, n = 15 mice; cfos+, n = 15 mice. Horizontal bars, mean. (**f-k**) Silencing tagged fully mature GNs during REM sleep. (**f**) Schematic representation of Jaws expression in DG granule neurons and virus injection. (**g**) Representative image of tagged DG granule neurons. The experiment was repeated in 3 mice with similar results. ml, molecular layer; gl, granular cell layer; sgz, subgranular zone. (**h**) Effect of light delivery on resting membrane potential and electrically evoked action potentials in Jaws+ GNs (n = 5 neurons). Scale bar: 10 mV and 100 ms. (**i**) Resting membrane potential, two-tailed paired *t*-test, $p = 0.86$. (**j**) Spike frequency, two-tailed paired *t*-test, $p = 0.009$. (h-j) The experiment was repeated in n = 5 neurons. (**k**) (left) Experience-dependent analysis of Jaws-GFP expression. (right) Jaws-GFP cell density (n = 3 mice, each group). TMX, tamoxifen; Dox, doxycycline; A, context A; C, context C; HC, home cage; REM, REM sleep; **, $p < 0.01$.

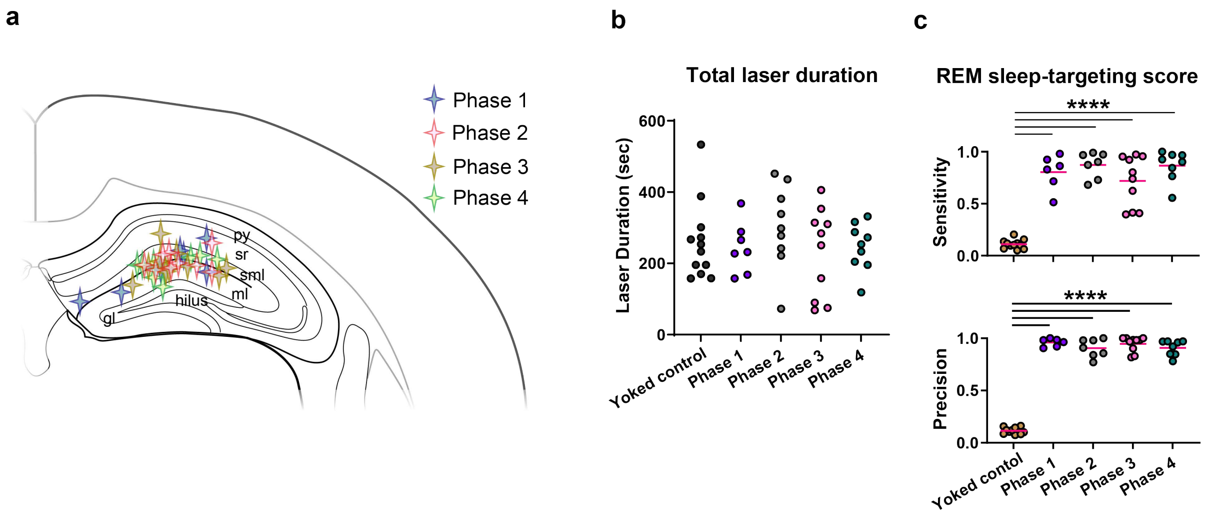

Supplementary Fig. 6. Theta phase-specific closed-loop optogenetic stimulation in REM sleep. (**a**) Location of the optic fiber and LFP electrode tips. gl, granular cell layer; gr, granule layer; ml, molecular layer; py, pyramidal layer; slm, stratum lacunosum-moleculare; sr, stratum radiatum. (**b**) Total laser duration, one-way ANOVA, $p = 0.65$. Yoked control, n = 12 mice; Phase 1, n = 7 mice; Phase 2, n = 9 mice; Phase 3, n = 10 mice; Phase 4, n = 9 mice. **c.** Sensitivity and precision of optogenetic stimulation during REM sleep, one-way ANOVA. Yoked control, n = 11 mice; Phase 1, n = 6 mice; Phase 2, n = 7 mice; Phase 3, n = 10 mice; Phase 4, n = 8 mice. Sensitivity: $p < 0.0001$, precision: $p < 0.0001$. Horizontal bars, mean. ****, $p < 0.0001$

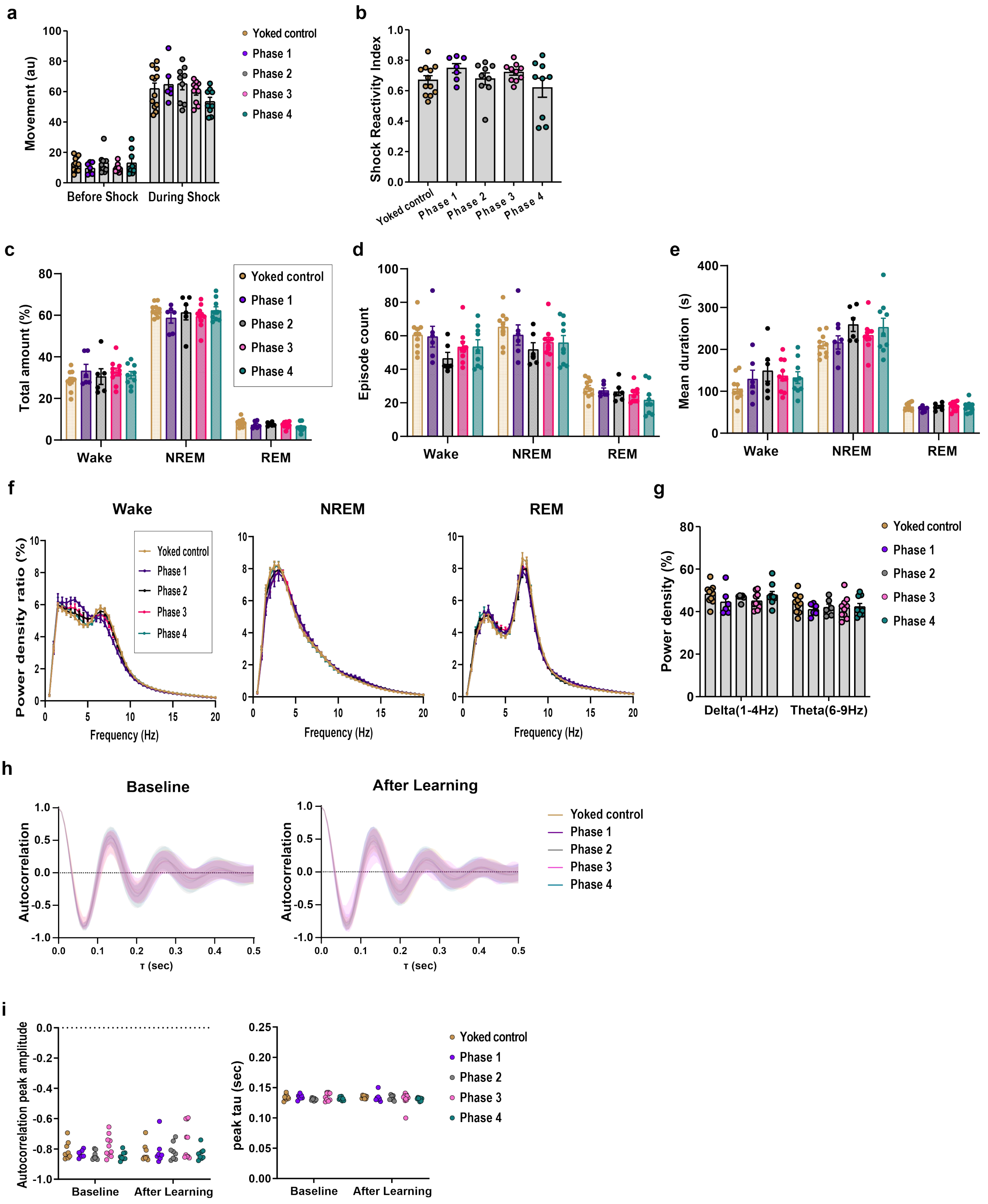

Supplementary Fig. 7. No difference in shock reactivity, sleep architecture, and DG theta oscillations among theta phase groups. (**a**) Movement of mice before and during foot shocks, two-way repeated measures ANOVA, $p = 0.076$. au, arbitrary units. (**b**) Shock reactivity index, one-way ANOVA, $p = 0.21$. Yoked control, n = 12 mice; Phase 1, n = 7 mice; Phase 2, n = 9 mice; Phase 3, n = 10 mice; Phase 4, n = 9 mice. (**c-g**) Sleep score under optogenetic silencing during REM sleep within 6 h after the conditioning session. (c) Total amount, two-way repeated measures ANOVA, $p = 0.61$. (**d**) Episode count, two-way repeated measures ANOVA, $p = 0.54$. (e) Mean duration, two-way repeated measures ANOVA, $p = 0.39$. (**f**) Fast Fourier transform analysis of EEG. (**g**) NREM sleep-delta (1-4 Hz) and REM sleep-theta (6-9 Hz) band,

two-way repeated measures ANOVA, p = 0.96. Yoked control, n = 10 mice; Phase 1, n = 6 mice; Phase 2, n = 6 mice; Phase 3, n = 10 mice; Phase 4, n = 9 mice. (**a-g**) Data are presented as mean values +/- SEM. (**h**) Mean autocorrelations of DG LFPs in REM sleep during memory consolidation. Yoked control, n = 221 epochs from 8 mice; Phase 1, n = 194 epochs from 7 mice; Phase 2, n =329 epochs from 8 mice; Phase 3, n = 321 epochs from 10 mice; Phase 4, n = 326 epochs from 8 mice. Shaded regions indicate standard deviation. (**i**) Autocorrelation first peak amplitude, two-way repeated measures ANOVA. p = 0.60. Autocorrelation peak tau, p = 0.55. Yoked control, n = 8 mice; Phase 1, n = 7 mice; Phase 2, n = 8 mice; Phase 3, n = 10 mice; Phase 4, n = 8 mice. Wake, wakefulness; NREM, Non-rapid eye movement sleep; REM, REM sleep; SEM, standard error of the mean.

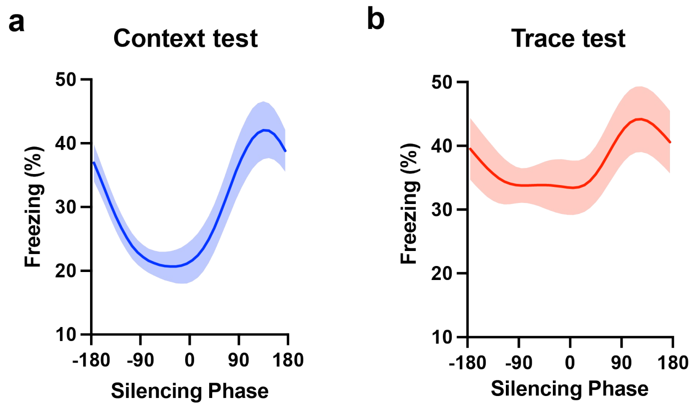

Supplementary Fig. 8. Predicted freezing behaviours resulting from the silencing of ABNs at different phases of the theta cycle. Freezing behaviour was modeled utilizing the histograms of light delivery corresponding to each mouse**.** To account for correlations between nearby theta phases and the high dimensionality of predictors, ridge regression was applied to estimate phase-dependent effects on freezing. The model incorporated 10°-binned stimulation histograms per mouse and was regularized via cross-validation. (**a, b**) Predicted freezing behaviours for the context and trace fear memory retrieval tests. Curves represent model-predicted freezing as a function of silencing phase, weighted by real stimulation patterns observed across animals. Shaded regions indicate 95% confidence intervals.

Supplementary Table 1. Linear regression summary for average neuron activity.

Parameter	Estimate	SE	DF	F	P-value (Random permutations)	Interpretation:
**Fixed effects:**						
Intercept	14.040	0.37	(1,4483)	1427.40	0.0001<	The intercept reflects average activity during the first exposure to context A.
Shock	-1.899	0.50	(1,4483)	7.52	0.002	Average calcium activity significantly decrease after shock.
REM	-4.390	1.15	(1,4483)	11.68	0.0001<	Average calcium activity significantly decrease during REM sleep.
Neuron category (GNs/ABNs)	-0.399	0.62	(1,4483)	0.44	0.456	Average calcium activity is not different between GNs and ABNs.
Time/24h	-1.671	0.16	(1,4483)	32.95	0.0001<	Average calcium activity decrease after repeated exposures to context A.
**Interaction with Neuron category:**						
Shock	-0.431	1.64	(1,4483)	0.06	0.763	Calcium activity decreases equally in ABNs and GNs after shock.
REM	-0.178	1.79	(1,4483)	0.03	0.805	Calcium activity decreases equally in ABNs and GNs during REM.
Time/24h	0.299	0.30	(1,4483)	0.68	0.345	Calcium activity decreases equally in ABNs and GNs after repeated exposures to the same context.

Model: Activity ~ 1 + Time*NeuronCategory + Shock*NeuronCategory + REM*NeuronCategory

Supplementary Table 2. Linear regression summary for PV correlations.

Parameter	Estimate	SE	DF	F	P-value	Interpretation
Fixed effects:						
Intercept	0.720	0.05	(1,208)	170.39	0.001<	Overall, PV are significantly correlated.
Shock	-0.060	0.05	(1,208)	1.44	0.232	PV correlations are not affected by shock experience.
REM	-0.270	0.06	(1,208)	17	0.001<	PV correlations decrease during REM sleep.
Neuron category (GNs/ABNs)	-0.010	0.08	(1,208)	0.02	0.87601	PV correlations are not different between ABNs and GNs.
Time/24h	-0.100	-0.1	(1,208)	55.2	0.001<	PV correlations significantly drift in time.
Interaction with Neuron category:						
Shock	-0.001	0.07	(1,208)	2.E-04	0.99	PV correlations are not affected by shock experience.
REM	0.220	0.09	(1,208)	5.7	0.018	REM PVs correlate more with the habituation context in ABNs than GNs.
Time/24h	0.070	0.02	(1,208)	11.3	0.001<	ABN PVs drift less over time than GNs.

Model: PV correlation ~ 1 + Shock*NeuronCategory + REM*NeuronCategory + NeuronCategory*Time

Supplementary Table 3. Statistics summary

Figure	Panel	Sample Size	Methods	Statistical values	Post hoc analysis
1	h	GNs, n = 363 neurons in 3 mice; ABNs, n = 136 neurons in 3 mice.	See Table S1 and S2.		
	k				
2	g	Jaws- (n = 10) and Jaws+ (n = 4) neurons	Two-way repeated measures ANOVA with Sidak's multiple comparisons	Condition x Neuron type: F (1, 12) = 51.14, p < 0.0001	Jaws-, p = 0.99; Jaws+, p < 0.0001
	h			Condition x Neuron type: F (1, 12) = 30.86, p = 0.0001	Jaws-, p = 0.630; Jaws+, p < 0.0001
	j	n = 14 mice	Two-tailed paired t-test	t(13) = 1.32, p = 0.21	
	l	n = 10 mice	Two-tailed paired t-test	t(9) = 2.20, p = 0.056	
3	b	cfos-, n = 15; cfos+, n = 14	Two-tailed unpaired t-test	t(27) = 3.40, p = 0.002	
	d	cfos-, n = 10; cfos+, n = 11		t(19) = 0.40, p = 0.69	
	f	cfos-, n = 10; cfos+, n = 12		t(20) = 0.90, p = 0.38	

	**h**	**cfos-, n = 13; cfos+, n = 14**		***t*(25) = 0.30, *p* = 0.77**	
**4**	**e**	**Yoked control, n = 12; Phase 1, n = 7; Phase 2, n = 9; Phase 2, n = 10; Phase 3, n = 9 mice**	**One-way ANOVA with Dunnett's multiple comparisons tests (all vs. yoked control).**	**Context test: *F*(4 , 42) = 3.489, *p* = 0.015; Trace test: *F*(4 , 42) = 2.679, *p* = 0.044**	**Context test: Yoked control vs Phase 1, *p* = 0.042; Trace test: *p* = 0.013**
**S2**	**a**	**GNs, n = 3, ABNs, n = 3 mice.**	**Two-way repeated measures ANOVA with Sidak's multiple comparisons**	**# of principal components × Neuron type: *F*(7, 28) = 0.748, *p* =0.63**	
	**b**	**GNs, n = 363 neurons in 3 mice; ABNs, n = 136 neurons in 3 mice.**	**See Table S4**		
	**c**	**GNs, n = 363 neurons in 3 mice; ABNs, n = 136 neurons in 3 mice.**	**Percentile bootstrap**	**Main effects:**<br><br>**Time = 0.001< ***<br>**Shock = 0.068 ns**<br>**Rem = 0.001< ***<br><br>**Interactions:**<br><br>**Time GN x ABNs = 0.001< ***<br>**Shock GN x ABNs = 0.9790 ns**	

				**REM GN x ABNs = 0.001< ***	
	**d**	**GNs, n = 3, ABNs, n = 3 mice.**	**Two-tailed unpaired t-test**	***t* = 1.103, *p* = 0.3317**	
	**e**	**GNs, n = 363 neurons in 3 mice; ABNs, n = 136 neurons in 3 mice.**	**Percentile bootstrap**	**Main effects:**<br><br>**Time = 0.001< ***<br>**Show = 0.0730 ns**<br>**Rem = 0.001< ***<br><br>**Interactions:**<br><br>**Time GN x ABNs = 0.001< ***<br>**Shock GN x ABNs = 0.823 ns**<br>**REM GN x ABNs = 0.001< ***	

	**f**	**ABNs, n = 3 mice.**	**ANOVA marginal tests**	**post-shock, $F(1,104) = 2.68$, $p = 0.105$; retrieval, $F(1,104) = 4.56$, $p = 0.036$.**	
**S4**	**a**	**cfos-, n = 15 mice; cfos+, n = 14 mice.**	**Two-way repeated measures ANOVA**	**Time x mouse group: $F(9, 243) = 0.25$, $p = 0.99$**	
	**b**		**Two-way repeated measures ANOVA**	**Time x mouse group: $F(1, 27) = 0.006$, $p = 0.94$**	
	**c**		**Two-tailed unpaired t-test**	**$t(27) = 1.49$, $p = 0.15$**	
	**e**	**cfos-, n = 10 mice; cfos+, n = 6 mice.**	**Two-way repeated measures ANOVA**	**Sleep stage x mouse group: $F(2,28) = 0.20$, $p = 0.82$**	
	**f**			**Sleep stage x mouse group: $F(2,28) = 0.17$, $p = 0.21$**	
	**g**			**Sleep stage x mouse group: $F(2,28) = 0.25$, $p = 0.78$**	
	**i**			**Sleep stage x mouse group: $F(1,14) = 2.03$, $p = 0.17$**	
**S5**	**b**	**cfos-, n = 14 mice; cfos+, n = 14 mice**	**Two-tailed unpaired t-test**	**$t = 2.92$, $p = 0.007$**	

	e	cfos-, n = 15 mice; cfos+, n = 15 mice.	Two-tailed unpaired t-test	t = 0.16, p = 0.87	
	i	n = 5 neurons	Two-tailed paired t-test	t = 6.13, p = 0.86	
	j		Two-tailed paired t-test	t = 4.71, p = 0.009	
S6	b	Yoked control, n = 12 mice; Phase 1, n = 7 mice; Phase 2, n = 9 mice; Phase 3, n = 10 mice; Phase 4, n = 9 mice.	One-way ANOVA	F(4 , 42) = 1.05, p = 0.65	
	c	Yoked control, n = 11 mice; Phase 1, n = 6 mice; Phase 2, n = 7 mice; Phase 3, n = 10 mice; Phase 4, n = 8 mice.	One-way ANOVA	Sensitivity: F(4 , 37) = 4.18, p < 0.0001; Precision: F(4 , 37) = 1.64, p < 0.0001	Sensitivity: Yoked control vs. Phase 1, Phase 2, Phase3, or Phase 4, p < 0.00001; Precision: Yoked control vs. Phase1, Phase 2, Phase3, and Phase4, p < 0.00001
S7	a	Yoked control, n = 12 mice; Phase 1, n = 7 mice; Phase 2, n = 9 mice; Phase 3, n = 10 mice; Phase 4, n = 9 mice.	Two-way repeated measures ANOVA	Condition x mouse group: F(4, 42) = 2.28, p = 0.076	
	b		One-way ANOVA	F(4, 42) = 1.79, p = 0.21	

	c	Yoked control, n = 10 mice; Phase 1, n = 6 mice; Phase 2, n = 6 mice; Phase 3, n = 10 mice; Phase 4, n = 9 mice.	Two-way repeated measures ANOVA	Sleep stage x mouse group: $F(8,72) = 0.79$, $p = 0.61$	
	d			Sleep stage x mouse group: $F(8,72) = 1.68$, $p = 0.54$	
	e			Sleep stage x mouse group: $F(8,72) = 1.08$, $p = 0.39$	
	g			Sleep stage x mouse group: $F(4,36) = 0.15$, $p = 0.96$	
	i	Yoked control, n = 8 mice; Phase 1, n = 7 mice; Phase 2, n = 8 mice; Phase 3, n = 10 mice; Phase 4, n = 8 mice.	Two-way repeated measures ANOVA	Autocorrelation first peak amplitude, Time x mouse group: F(4,36) = 0.69, $p = 0.60$. Autocorrelation peak tau, Time x mouse group: F(4,36) = 0.78, $p = 0.55$	

Supplementary Table 4. Linear regression summary for PV correlations, including number of neurons as covariate.

**Parameter**	**Estimate**	**SE**	**DF**	**F**	**P-value**	**Interpretation:**
**Fixed effects:**						
**Intercept**	**0.591**	**0.08**	**(1, 207)**	**1.93**	**0.0001<**	**Overall, PV are significantly correlated.**
**Shock**	**-0.058**	**0.05**	**(1, 207)**	**1.55**	**0.21433**	**PV correlations are not affected by shock experience.**
**REM**	**-0.266**	**0.06**	**(1, 207)**	**16.85**	**0.0001<**	**PV correlations decrease during REM sleep.**
**Neuron category (GNs/ABNs)**	**0.248**	**0.15**	**(1, 207)**	**2.70**	**0.10212**	**PV correlations are not different between ABNs and GNs.**
**Time/24h**	**-0.104**	**0.01**	**(1, 207)**	**55.13**	**0.0001<**	**PV correlations significantly drift in time.**
**Size**	**0.003**	**0.00**	**(1, 207)**	**3.73**	**0.054917**	**PV correlations are not different between the # of ABNs and GNs.**
**Interaction with Neuron category:**						
**Shock**	**-0.001**	**0.07**	**(1,207)**	**0.0002**	**0.98874**	**PV correlations are not affected by shock experience.**
**REM**	**0.218**	**0.09**	**(1, 207)**	**5.65**	**0.018416**	**REM PVs correlate more with the habituation context in ABNs than GNs.**
**Time/24h**	**0.066**	**0.02**	**(1, 207)**	**11.28**	**0.001<**	**ABN PVs drift less over time than GNs.**

Model: PV correlation ~ 1 + #neurons+ Shock*NeuronCategory + REM*NeuronCategory + NeuronCategory*Time

139
