# Supplementary material for: Transient reactivation of small ensembles of adult-born neurons during REM sleep supports memory consolidation in mice
Source: Nat Commun. 2025 Aug 5;16:7210. doi: 10.1038/s41467-025-62554-8 (PMC12325634; doi:10.1038/s41467-025-62554-8)
Supplement: Supplementary file 2 — Description of Additional Supplementary Files [file 41467_2025_62554_MOESM2_ESM.pdf]

Description of Additional Supplementary Files:

Supplementary Movie 1: ABN Ca^{2+} imaging recording from postS and a subsequent REM episode, related to Fig. 1. The video shows a ~30-s recording of young ABNs, played at 3× speed. Colored contours highlight three neurons that were active during the postS sessions and reactivated during a subsequent REM episode.
