# Supplementary material for: Transient reactivation of small ensembles of adult-born neurons during REM sleep supports memory consolidation in mice
Source: Nat Commun. 2025 Aug 5;16:7210. doi: 10.1038/s41467-025-62554-8 (PMC12325634; doi:10.1038/s41467-025-62554-8)
Supplement: Supplementary file 4 — Reporting Summary [file 41467_2025_62554_MOESM4_ESM.pdf]

nature portfolio

Corresponding author(s): Masanori Sakaguchi

Last updated by author(s): Jul 4, 2025

Reporting Summary

Nature Portfolio wishes to improve the reproducibility of the work that we publish. This form provides structure for consistency and transparency in reporting. For further information on Nature Portfolio policies, see our Editorial Policies and the Editorial Policy Checklist.

Statistics

For all statistical analyses, confirm that the following items are present in the figure legend, table legend, main text, or Methods section.

n/a	Confirmed	
☐	☒	The exact sample size (n) for each experimental group/condition, given as a discrete number and unit of measurement
☐	☒	A statement on whether measurements were taken from distinct samples or whether the same sample was measured repeatedly
☐	☒	The statistical test(s) used AND whether they are one- or two-sided<br>*Only common tests should be described solely by name; describe more complex techniques in the Methods section.*
☐	☒	A description of all covariates tested
☐	☒	A description of any assumptions or corrections, such as tests of normality and adjustment for multiple comparisons
☐	☒	A full description of the statistical parameters including central tendency (e.g. means) or other basic estimates (e.g. regression coefficient) AND variation (e.g. standard deviation) or associated estimates of uncertainty (e.g. confidence intervals)
☐	☒	For null hypothesis testing, the test statistic (e.g. F, t, r) with confidence intervals, effect sizes, degrees of freedom and P value noted<br>*Give P values as exact values whenever suitable.*
☒	☐	For Bayesian analysis, information on the choice of priors and Markov chain Monte Carlo settings
☒	☐	For hierarchical and complex designs, identification of the appropriate level for tests and full reporting of outcomes
☒	☐	Estimates of effect sizes (e.g. Cohen's d, Pearson's r), indicating how they were calculated

Our web collection on statistics for biologists contains articles on many of the points above.

Software and code

Policy information about availability of computer code

Data collection	Freezing data were collected using Freezeframe version 4.104 (Med Associates).<br>EEG/EMG/LFP were recorded using SleepSignRecorder or Vital Recorder (Kissei Comtec).<br>Automatic sleep staging and optic stimulation were performed with UTSN-L2 (https://github.com/tarotez/sleepstages) and SleepSignRecorder (Kissei Comtec).<br>In vivo calcium imaging data were collected with nVoke acquisition software (Inscopix, USA).<br>Electrophysiological signals were recorded using Igor Pro (WaveMetrics).
Data analysis	Freezing behavior was measured using Freezeframe version 4.104 (Med Associates).<br>Calcium signals were extracted and tracked across sessions utilizing CaliAli release v1-beta (https://www.biorxiv.org/content/10.1101/2023.05.19.540935v1).<br>Statistical analysis was performed using GraphPad Prism version 9 (GraphPad Software, USA), custom scripts in MATLAB (MathWorks, USA) and R.

For manuscripts utilizing custom algorithms or software that are central to the research but not yet described in published literature, software must be made available to editors and reviewers. We strongly encourage code deposition in a community repository (e.g. GitHub). See the Nature Portfolio guidelines for submitting code & software for further information.

Data

Policy information about availability of data

All manuscripts must include a data availability statement. This statement should provide the following information, where applicable:

- Accession codes, unique identifiers, or web links for publicly available datasets
- A description of any restrictions on data availability
- For clinical datasets or third party data, please ensure that the statement adheres to our policy

Data availability
No unique reagents were generated in this study. Data for LFP and phase-specific silencing study is available at Mendeley Data [https://doi.org/10.17632/f4646v4fp8.1]. All detailed statistics are included in the Supplementary information. All other data supporting this study are available from the corresponding authors. Source data are provided with this paper.

Code availability
The datasets and custom code used to perform the non-trivial quantitative analyses are available on GitHub [https://github.com/vergaloy/Srinivasan_Koyanagi_2025]. A preserved snapshot of the code at the time of publication has also been archived on Zenodo [https://zenodo.org/records/15803872].

Research involving human participants, their data, or biological material

Policy information about studies with human participants or human data. See also policy information about sex, gender (identity/presentation), and sexual orientation and race, ethnicity and racism.

Reporting on sex and gender	This study did not involve human participants or human data.
Reporting on race, ethnicity, or other socially relevant groupings	N/A
Population characteristics	N/A
Recruitment	N/A
Ethics oversight	N/A

Note that full information on the approval of the study protocol must also be provided in the manuscript.

Field-specific reporting

Please select the one below that is the best fit for your research. If you are not sure, read the appropriate sections before making your selection.

- [x] Life sciences
- [ ] Behavioural & social sciences
- [ ] Ecological, evolutionary & environmental sciences

For a reference copy of the document with all sections, see nature.com/documents/nr-reporting-summary-flat.pdf

Life sciences study design

All studies must disclose on these points even when the disclosure is negative.

Sample size	No statistical method was used to pre-determine sample sizes. We determined sample size based on the previous literature (Kumar et al., Neuron, 107 552-565.e1-e10, 2020). The number of animals in each experimental group are standard sample sizes in this field.
Data exclusions	No data were excluded after obtaining primary endpoint data.
Replication	The number of biologically independent samples is provided in the figure legends for each experiment, and all experiments include appropriate controls. The data of core finding (Figure 3a-b) was replicated in an independent experiment (Extended Data Fig. 3a-b).
Randomization	Mice were randomly assigned to each groups, and experiments were run by alternating between each groups.
Blinding	All behavioral experiments were conducted and analyzed by researchers who were blind to the subject's cohort assignment.

Reporting for specific materials, systems and methods

We require information from authors about some types of materials, experimental systems and methods used in many studies. Here, indicate whether each material, system or method listed is relevant to your study. If you are not sure if a list item applies to your research, read the appropriate section before selecting a response.

Materials & experimental systems

n/a	Involved in the study	
	x	Antibodies
x		Eukaryotic cell lines
x		Palaeontology and archaeology
	x	Animals and other organisms
x		Clinical data
x		Dual use research of concern
x		Plants

Methods

n/a	Involved in the study	
x		ChIP-seq
x		Flow cytometry
x		MRI-based neuroimaging

Antibodies

Antibodies used	rabbit polyclonal anti-GFP primary antibodies (1:250, Thermo Fisher Scientific Cat# A-11122, RRID:AB_221569), anti-rabbit HRP secondary antibodies (1:500, Jackson ImmunoResearch Labs Cat# 711-036-152, RRID:AB_2340590), and streptavidin Alexa-488 (1:500, ImmunoResearch Labs Cat# 016-540-084, RRID:AB_2337249).
Validation	Each antibody is commercially available. The specificity of these antibodies was validated by the manufacturers. Validation profiles for each antibody can be found in the links provided.<br><br>rabbit polyclonal anti-GFP primary antibodies (1:250, Thermo Fisher Scientific Cat# A-11122, RRID:AB_221569: https://www.thermofisher.com/antibody/product/GFP-Antibody-Polyclonal/A-11122)<br><br>anti-rabbit HRP secondary antibodies (1:500, Jackson ImmunoResearch Labs Cat# 711-036-152, RRID:AB_2340590: https://www.jacksonimmuno.com/catalog/products/711-036-152)<br><br>streptavidin Alexa-488 (1:500, ImmunoResearch Labs Cat# 016-540-084, RRID:AB_2337249: https://www.jacksonimmuno.com/catalog/products/016-540-084)

Animals and other research organisms

Policy information about studies involving animals; ARRIVE guidelines recommended for reporting animal research, and Sex and Gender in Research

Laboratory animals	Wild-type C57BL/6J mice, pNestin-CreERT2 (nestin mice, The Jackson Laboratory, JAX:016261, RRID:IMSR_JAX:016261)18, Ai94(TITL-GCaMP6s)-ROSA26-ZtTA (TRE-LSL-GCaMP6s) (GCaMP6s mice, The Jackson Laboratory, JAX:024112, RRID:IMSR_JAX:024112), Ai79D (Rosa26-TRE-loxP-stop-loxP-Jaws-GFP)(jaws mice, The Jackson Laboratory, JAX:023529, RRID:IMSR_JAX:023529), and Ai39(Rosa26-pCAG-LSL-eNpH3.0-YFP)(halo mice, The Jackson Laboratory, JAX:014539, RRID:IMSR_JAX:014539) were purchased from Jackson Laboratory. cfos-tTA (cfos mice) were derived from the Mutant Mouse Regional Resource Center (stock no. 031756-MU, RRID:MMRRC_ 031756-MU). All transgenic mice were backcrossed in a C57BL6/J background more than 10 times. All transgenes were kept as heterozygotes in the chromosome used for the experiments to avoid possible complications of overexpressing Cre recombinase, tTA, and loss of the Rosa allele. All mice were maintained at an ambient temperature of 23.5 ± 2.0°C with 50±5% humidity under a 12-h light/dark cycle (9 am to 9 pm) with ad libitum access to food and water in accordance with institutional guidelines.
Wild animals	Wild animals were not used in the study.
Reporting on sex	Both female and male mice were used in this study, except for Figure 4. Only female mice were used in Figure 4 due to limitations in animal availability at that time.
Field-collected samples	The study did not involve samples collected from the field.
Ethics oversight	All animal experiments were approved by the University of Tsukuba Institutional Animal Care and Use Committee (Animal experimental approval# 23-237, Gene recombination experiment approval# 210122).

Note that full information on the approval of the study protocol must also be provided in the manuscript.

Plants

Seed stocks	N/A
Novel plant genotypes	N/A
Authentication	N/A
